# Nectar in Plant–Insect Mutualistic Relationships: From Food Reward to Partner Manipulation

**DOI:** 10.3389/fpls.2018.01063

**Published:** 2018-07-19

**Authors:** Massimo Nepi, Donato A. Grasso, Stefano Mancuso

**Affiliations:** ^1^Department of Life Sciences, University of Siena, Siena, Italy; ^2^Department of Chemistry, Life Sciences and Environmental Sustainability, University of Parma, Parma, Italy; ^3^Department of Agrifood Production and Environmental Sciences, University of Florence, Florence, Italy

**Keywords:** nectar, partner manipulation, secondary compounds, mutualistic relationships, exploitation, plant–animal interactions

## Abstract

It has been known for centuries that floral and extra-floral nectar secreted by plants attracts and rewards animals. Extra-floral nectar is involved in so-called indirect defense by attracting animals (generally ants) that prey on herbivores, or by discouraging herbivores from feeding on the plant. Floral nectar is presented inside the flower close to the reproductive organs and rewards animals that perform pollination while visiting the flower. In both cases nectar is a source of carbon and nitrogen compounds that feed animals, the most abundant solutes being sugars and amino acids. Plant–animal relationships involving the two types of nectar have therefore been used for a long time as text-book examples of symmetric mutualism: services provided by animals to plants in exchange for food provided by plants to animals. Cheating (or deception or exploitation), namely obtaining the reward/service without returning any counterpart, is however, well-known in mutualistic relationships, since the interacting partners have conflicting interests and selection may favor cheating strategies. A more subtle way of exploiting mutualism was recently highlighted. It implies the evolution of strategies to maximize the benefits obtained by one partner while still providing the reward/service to the other partner. Several substances other than sugars and amino acids have been found in nectar and some affect the foraging behavior of insects and potentially increase the benefits to the plant. Such substances can be considered plant cues to exploit mutualism. Recent evidence motivated some authors to use the term “manipulation” of animals by plants in nectar-mediated mutualistic relationships. This review highlights the recent background of the “manipulation” hypothesis, discussing it in the framework of new ecological and evolutionary scenarios in plant–animal interactions, as a stimulus for future research.

## Introduction

Mutualistic inter-species relationships, i.e., relationships in which interacting species reciprocate benefits received, are very common in all kingdoms of living organisms since virtually every species is involved in one or more such relationships. Mutualism has a pivotal role in the functioning of all current ecosystems and in key events of the evolutionary history of life on our planet, such as the evolution of eukaryotic cells, colonization of land by plants and the radiation of angiosperms (Bronstein et al., 2006; Douglas, 2010; Bronstein, 2015).

Mutualisms have often been reported as evidence of the ancient classical theory of “balance of nature” that is rooted in Greek philosophy and mythology. According to this theory natural systems tend to remain in a stable equilibrium where natural forces prevent species from becoming too abundant or becoming extinct (Egerton, 1973). Mutualism, regarded as reciprocal cooperation between species, was therefore perfectly framed in this theory. The balance of nature was challenged by the evolutionary theory based on natural selection elaborated by Darwin (1859), according to which “natural selection cannot possibly produce any modification in a species exclusively for the good of another species.” From a more recent evolutionary point of view, mutualistic relationships hide an apparent paradox since each species tends to maximize its own fitness when interacting with another and unrelated partners may have conflicts of interests (Sachs, 2015). These conflicts challenge the maintenance of mutualisms and selection may favor exploitation or the abandonment of such relationships. However, possible conflicts can be managed and mutualism stabilized in different ways, from special rewards for cooperatives and sanctions for cheaters to strict specificity in partner choice (Douglas, 2008, 2010). An additional possibility is to rely on some form of coercion/manipulation of the partner without disrupting the mutually beneficial outcomes of the relationship (Grasso et al., 2015; Heil, 2015a). From this point of view, mutualisms can best be regarded as reciprocally exploitative interactions that provide a net benefit to both parties. The net effect to each partner is highest when the benefit is maximized in relation to investment (Bronstein, 2001 and references therein).

Plants are involved in a myriad of mutualistic interactions with very diverse organisms such as bacteria, fungi and animals. Mutualisms with bacteria (nitrogen-fixing bacteria) and fungi (mycorrhiza) increase nutrient uptake by plants as well as providing organic matter and a suitable ecological niche to the heterotrophic counterpart. Since plants are anchored to the ground and have limited possibility of movements, the benefits they receive in mutualistic interactions with animals, and especially insects, arise from insects’ ability to cover long distances. The animal’s ability to move is involved in two processes invaluable for plant survival: dispersal of propagules, mainly pollen and seeds, and indirect defense against herbivory (Schoonhoven et al., 2005; Rico-Gray and Oliveira, 2007).

Pollen, the male gametophyte of seed plants containing the male gametes, needs to be transported from the anther to the stigma of a compatible carpel, a process called pollen dispersal or pollination that is the first step toward fertilization in all seed plants. According to a recent global estimate, 87.5% of all angiosperms are pollinated by animals (Ollerton et al., 2011) and a significant fraction of gymnosperms are ambophilous, i.e., pollinated by wind and by animals as well (Nepi et al., 2017). Insects are the most numerous and diverse animals involved in pollination (Ollerton, 2017). Besides its importance from an ecological and evolutionary perspective, pollination has great economic value: more than one third of human food resources are derived from insect pollination and about 1500 crop species worldwide are pollinated by insects, so the estimated economic value of this ecosystem service adds up to $360 billion (Hanley et al., 2015).

Being sessile and having limited movements, plants have developed an array of defense strategies against predation by herbivores (Schoonhoven et al., 2005). Direct defenses involve morphological and chemical cues that discourage herbivores from feeding on a plant. Plants may also engage in mutualistic relationships with arthropods, such as ants, wasps, spiders, mites, and parasitoids, that patrol the plant and deter or even kill herbivores (Arimura et al., 2005; Dicke and Baldwin, 2010; Heil, 2015a). The plant defends itself indirectly by attracting an animal “body-guard” via a tritrophic interaction (Heil, 2008). Indirect defense based on mutualism with ants, on which we focus in this review, has wide phylogenetic and geographic distribution, although the highest level of complexity and coadaptation of plant–ant relationships is reached in angiosperms of tropical and subtropical regions (Hölldobler and Wilson, 1990; Heil and McKey, 2003; Rico-Gray and Oliveira, 2007; Ness et al., 2010). Indirect defense involving ants is very efficient and has also evolved outside the plant kingdom: aphids (Hemiptera, Aphididae) as well as caterpillars of certain species of blue butterflies (Lepidoptera: Lycaenidae) are protected indirectly by ants against their predators (Nepi, 2017 and references therein).

**FIGURE 1:**
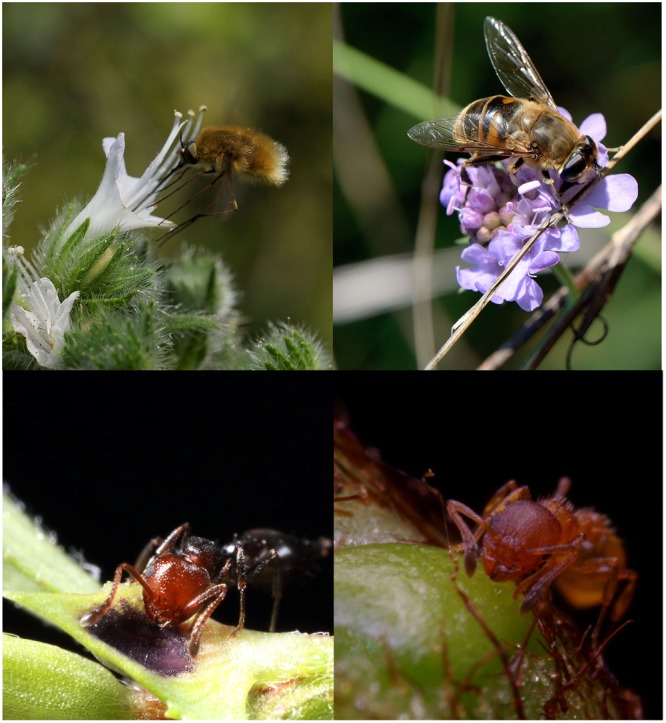
Arthropods feeding on floral nectar (FN) **(top)** and extra-floral nectar (EFN) **(bottom)**. *Bombylius* sp. probing for nectar in a flower of *Echium italicum* (top left; picture by Sara Mancini, Department of Environmental Sciences, University of Siena); *Eristalis tenax* foraging for nectar on the flowers of *Scabiosa* sp. (top right; picture by MN, Department of Environmental Sciences, University of Siena); *Crematogaster scutellaris* feeding on EFN produced by stipular nectaries of *Vicia sativa* (bottom left; picture by Daniele Giannetti and DG, Myrmecology Lab, University of Parma); *Temnothorax* sp. collecting nectar from a foliar nectary of *Pteridium aquilinum* (bottom right; picture by Daniele Giannetti and DG, Myrmecology Lab, University of Parma).

Irrespective of the type of mutualism, whether for pollination or indirect defense, the benefit earned by the animal is generally a food resource produced by the plant, in most cases nectar. Nectar involved in mutualistic relationships with pollinators is called floral nectar (FN, **Figure 1**) since it is produced by organs (nectaries), usually inside the flower close to the reproductive organs, whereas nectar involved in indirect defense is generally offered in the vegetative part of the plant and is known as extra-floral nectar (EFN, **Figure 1**). Most insect pollinated angiosperms produce FN as the main primary floral attractant and their floral nectaries vary widely in position, shape and structure (Galetto, 2007). EFN is reported in about 4000 plant species (with estimations up to 8000 plant species), which are distributed among 457 independent lineages and living in a wide variety of tropical, subtropical and temperate habitats (Marazzi et al., 2013; Weber and Keeler, 2013). Both types of nectar, being sugary water-based acellular secretions, are easily collected, ingested, digested and absorbed by an extraordinary variety of animals, making it a ready-to-use energy source (Nicolson, 2007). Thus for 100s of years nectar-based plant–pollinator relationships (and subsequently plant–ant mutualism) have been reported as examples of symmetric mutualism: services provided by animals to plants in exchange for food provided by plants to animals. These cooperative relationships fit into the “balance of nature” theory, a perspective that still permeates modern ecology textbooks and papers that frequently refer to nectar as a “reward” for pollinators or plant defenders, attributing an exclusively cooperative meaning to such interactions. However, mutualisms may also be established on a selfish basis, limited by costs and driven by conflicts of interest between partners (Bronstein et al., 2006). Conflicts of interest between interacting partners clearly characterize nectar-mediated plant–animal interactions: plants target efficient service (pollination or indirect defense) by nectarivores at the lowest possible cost (thus minimizing the quantity of nectar they produce), while animals are interested in obtaining good quality food in sufficient quantity (nectar) irrespective of whether pollination or indirect defense of the plant is involved. For example, animals can detect humidity gradients over flowers that enable them to assess the amount of FN without probing the flowers and touching the reproductive organs (von Arx et al., 2012). In this scenario, selection would tend to favor exploitation of mutualism (Sachs, 2015) and examples of pure exploiters are well-known on both sides. Although orchids are insect pollinated, about one third do not produce any kind of food (Ackerman, 1986). The flowers of these nectarless orchids rely on several types of mimicry to attract insects, including specific resemblance to flowers of nectar-producing species (Johnson, 2000; Jersáková et al., 2006). Insects are not able to discriminate the flowers of the two species and visit the flowers of the nectarless orchid by mistake (Johnson, 2000). On the other hand, nectar robbing by insects that do not perform pollination has been known since the early observations of bumblebees stealing nectar from flowers of *Pentstemon, Antirrhinum, Stachys*, and *Salvia* (Darwin, 1841). Pure exploiters are also known in plant–animal relationships involving indirect defense. For example, ants of the genus *Cataulacus* (*C. mckeyi*) exploit the EFN of *Leonardoxa africana* without protecting the plants from herbivores (Gaume and Mckey, 1999). Nonetheless, the costs and benefits for both partners associated with cheating are not always univocal and cheating may sometimes not have detrimental effects (Maloof and Inouye, 2000, see below). Beyond pure exploitation or cheating, relationships with mutually beneficial outcomes are even subject to selective pressure to maximize the benefits obtained by one partner while still providing the reward/service to the other partner. These strategies can be considered more nuanced styles of exploitation than pure cheating, since the mutualism has costs for both partners. It was recently demonstrated that such interactions are (or may be) mediated by specific nectar compounds (Wright et al., 2013; Heil et al., 2014; Nepi, 2014; Grasso et al., 2015; Baracchi et al., 2017). Since most of these compounds modify insect physiology and behavior, this recent evidence has motivated researchers to regard them as a form of “manipulation” of animals by plants, namely mutualisms with a coercive component.

The aim of this review is to disentangle the complex ecological and evolutionary scenario recently revealed in nectar-mediated plant–animal interactions by considering mutualism, cheating and exploitation in a wider ecological framework and by analyzing the background of the “manipulation” hypothesis. Manipulative strategies seem to be more common in mutualistic relationships than was previously thought and they can be regarded as adaptations to counteract the temptation to cheat with the ultimate effect of stabilizing the mutualism (Heil, 2015b).

## Nectar Production Is Costly for Plants

In the evolutionary history of plants, nectar first appeared in pteridophytes (Schuettpelz and Pryer, 2008). Nectaries of pteridophytes are located on the fronds and are known as foliar nectaries (Koptur et al., 2013). They can therefore be considered topographically analogous to the EFNs of angiosperms. According to the exploitation hypothesis (*sensu* Del-Claro et al., 2016) derived from early physiological studies (Nieuwenhuis von Üxküll-Güldenband, 1907; Zimmermann, 1932), nectar was secreted as a “waste product” of excess carbohydrates, a theory that was probably inspired by Darwin (1859), since in On the origin of species, he wrote “certain plants excrete sweet juice, apparently for the sake of eliminating something injurious from the sap.” This hypothesis was recently re-considered by De la Barrera and Nobel (2004). Speculating on the origin of nectar secretion, these authors proposed two alternative hypotheses. The “sugar excretion” hypothesis proposes that nectar production arose to remove excess solutes supplied by the phloem and is triggered by intense transpiration of developing organs, somehow similar to the original physiological hypothesis. The “leaky phloem” hypothesis argues that nectar secretion is a leakage of phloem solution, resulting from the structural weakness of developing tissues exposed to high pressure in the phloem (De la Barrera and Nobel, 2004). Both hypotheses are in contrast with the different composition of EFN (and even FN) and phloem sap found in some extant species (Keeler, 1977; Nicolson and Thornburg, 2007) but they could be in line with the early appearance of nectar in pteridophytes. Today foliar nectaries are found in two clades of pteridophytes (Marattiales and leptosporangiate ferns) dating to the Palaeozoic (Schuettpelz and Pryer, 2008). Since ants originated in the early Cretaceous, 135–115 Mya (Ward, 2007), EFN probably initially had a function not involving ants. The latter began to exploit the sugary secretion soon after their origin (Nepi et al., 2009). Foliar nectar of extant ferns can be considered functionally similar to the EFN of angiosperms, since it may be involved in recruiting ants that protect against herbivores, although this function is more variable and controversial than in angiosperms (Koptur et al., 2013). Angiosperms, which evolved and radiated in the early-middle Cretaceous, reinforced nectar-mediated interactions with animals by adjusting the chemical, physiological and phenological traits of nectar in relation to the needs of new co-evolving insect groups (Nepi et al., 2017). These new adaptations presumably imply a higher cost of nectar production than for the “leaky phloem” or “sugar excretion” hypotheses of “early” nectar. Estimates of FN production costs in extant species in terms of daily photosynthate vary from 3.3% in short-lived flowers to 37% in long-lived flowers (Southwick, 1984). A trade-off between nectar production and plant growth-reproduction has also been demonstrated (Pyke, 1991). Although such estimates are not available for EFN, a cost for its production can be assumed, since there is evidence that FN and EFN are produced by the same general mechanisms (Heil, 2015a) and the composition of both types may differ from that of phloem sap (Keeler, 1977; Nicolson and Thornburg, 2007 and references therein). Indirect evidence of the cost of EFN and FN is that it is often reabsorbed if not consumed, and the carbohydrates are presumably allocated for other purposes (Búrquez and Corbet, 1991; Nepi and Stpiczyńska, 2008; Escalante-Pérez et al., 2012).

## Uncertainty of Benefits in Nectar-Mediated Interactions

The classical view of plant–animal interactions considers the services of pollination and indirect defense to be the main benefits for plants producing FN and EFN, respectively, whereas the animal counterpart obtains nutritious food. Nectar-producing plants have a higher probability of attracting insects that accomplish pollination and thus a higher probability of producing seeds. For example in several species exhibiting variability in nectar production between individuals, it was revealed that high nectar availability favors pollinator attraction, promoting floral visits and reproductive output, whereas decreased seed set was found in low nectar-producing individuals (Real and Rathcke, 1991; Galetto and Bernardello, 2004; Brandenburg et al., 2012; Cruz-Neto et al., 2015 and references therein).

In EFN-bearing plants, various ant-exclusion experiments clearly demonstrated (in most cases but not in all, see Sanz-Veiga et al., 2017 and references therein) an indirect function of EFN in protecting against herbivores and thus increasing plant fitness (reviewed in Heil, 2015a). The direct link between EFN, ants and plant defense is highlighted by induction of extra-floral nectary activity: herbivore damage may increase EFN production, increasing ant recruitment and raising protection (Heil, 2015a; Del-Claro et al., 2016 and reference therein). Interestingly, the activity of herbivores may affect interactions with pollinators (Adler et al., 2006; Rusman et al., 2018), so that ants defending plants against herbivores indirectly also protect plant interactions with pollinators, further improving plant fitness.

On the animal side, relatively few studies have assessed the benefits of feeding on nectar. Beyond being an energy source for insect flight by virtue of its high sugar content, specific nectar components are recognized as beneficial for pollinating insects. For example, amino acid-rich nectar improves butterfly fecundity (Mevi-Schütz and Erhardt, 2005). Proline, one of the more common and abundant amino acids in FN (Baker and Baker, 1983a), is required by honey bees for egg lying and increases the size of their hypopharyngeal gland acini (Darvishzadeh et al., 2015), organs that produce royal jelly. Oxidative degradation of proline is also used by some bees and wasps to fuel their flight (Carter et al., 2006; Teulier et al., 2016). Certain secondary metabolites detected in FN (such as gelsemine, anabasine, and nicotine) may benefit pollinators by increasing their resistance to parasites and pathogens (Roy et al., 2017; Stevenson et al., 2017 and references therein). EFN is reported to be a valuable resource for certain ant species, since it increases individual and colony growth rate and survival (Byk and Del-Claro, 2011). Increased survival and growth rate have also been reported for non-ant consumers, such as spiders and parasitoids, which additionally showed increased egg production and increased parasitization rate, respectively, after feeding on EFN (Heil, 2015b and reference therein).

In this general framework, the outcome of these interactions is highly conditional, varying in space and time and according to the species involved, partner behavior, environmental constraints and ecological context (Menzel et al., 2014; Hoeksma and Bruna, 2015; Del-Claro et al., 2016). The plants involved in nectar-mediated interactions with animals therefore pay the cost of nectar production for benefits that may not accrue.

For example, bumblebees (common pollinators of cultivated and native plants) sometimes rob nectar from flowers with long tubular corollas or spurs where the nectar is inaccessible (Inouye, 1983; Irwin et al., 2010). Robbing is a foraging strategy by which insects obtain nectar without contacting the reproductive organs of the flower and performing pollination. It is done by biting the base of a flower close to the nectar reservoir (primary robbing) or by exploiting perforations made by other animals (secondary robbing). Surprisingly, bumblebees rob FN in species that they could pollinate legitimately. This particular behavior could be due to obstacles to reaching nectar in the conventional way: hairs and structural barriers that hamper nectar access can often be avoided by unconventional routes to the nectar. Alternatively, large sticky pollen grains, which adhere to the body of insects visiting flowers in the conventional way can be bothersome and therefore promote nectar theft. It has been demonstrated that bumblebees finding robbed flowers significantly increased their behavior as primary robbers although they previously behaved as legitimate pollinators (Leadbeater and Chittka, 2008). Interaction with other bumblebees that practice secondary robbing can turn a legitimate forager bumblebee into a secondary robber. Since other insect species may also make holes to steal nectar, it seems likely that such interactions may involve heterospecific individuals (Leadbeater and Chittka, 2008 and references therein). Thus it appears that nectar robbing behavior may spread by social transmission through a community of insects, with plausibly negative effects on the plant community. However, robbers are not always detrimental, as frequently assumed for cheating since this term has a negative significance for humans. The frequency of negative, neutral and positive effects was actually equal in 18 studies that measured the effect of robbing on seed set (Maloof and Inouye, 2000) and the same robber species can have different effects on the reproductive success of distinct plant species (Bergamo and Sazima, 2018). Cheaters and robbers such as bumble bees and carpenter bees are also in some cases reported to be pollinators of the flowers they rob (Sampson et al., 2004; Zhu et al., 2010; Singh et al., 2014).

Insect behavior and ecological context are also responsible for indirect costs that may arise from ant-plant mutualism mediated by EFN. As nectar sources could be vital for individual nutrition and colony survival, some ants may also forage FN (Santos et al., 2014) but are generally not considered good pollinators because their metapleural glands produce anti-bacterial and anti-fungal secretions that disrupt the normal function of pollen grains (Peakall et al., 1990). They may have a negative effect on plant–pollinator mutualism by decreasing the quantity of FN available. Ants may also feed on pollen, reducing flower fertilization (Del-Claro et al., 2016). Furthermore, flower-visiting ants may deter and/or prey on pollinators, although this does not seem to affect the plant’s fruiting (Assunção et al., 2014). In other cases, ant behavior may have a direct and extremely detrimental effect on plant reproduction. The ants *Allomerus cf. demerarae* and *Crematogaster nigriceps* “castrate” their host plants, the former removing flowers from *Cordia nodosa* and the latter pruning axillary shoots bearing the inflorescences of *Acacia drepanolobium* (Young et al., 1997; Yu and Pierce, 1998). In this way they promote vegetative growth of the host plant, which thus produces more domatia and EFN, to the detriment of plant reproduction. Nonetheless, this behavior may have a positive effect on plant fitness in the long term, since young plants can be preferred by ants that strongly promotes their survival. Once older, plants can be colonized by other ant species that do not sterilize them allowing their reproduction (Palmer et al., 2010). The overall effect may be an increase in plant fitness.

## Insect Foraging Activities Are Affected by Plants Through Nectar Traits

The few examples reported above show that nectar-foraging behavior of animals may be unpredictable and highly variable, exposing nectar producing plants to the risk of not receiving any real benefit as a counterpart for the expense of nectar production. Selection can therefore be expected to favor strategies to counteract this risk. Plants have several ways of affecting the behavior of nectar foragers and study of these effects led to the first hypotheses about nectar-based manipulation of insects by plants (Biernaskie and Cartar, 2004; Pyke, 2016). The studies were almost exclusively focused on relationships between FN and pollinators, but the manipulation hypothesis was recently extended to EFN and ants (Grasso et al., 2015).

### Plants Affect Pollinator Foraging Behavior by Providing a Highly Variable Nectar Source

The reproductive success of plant species that rely on pollination by insects is determined by insect foraging activity. The behavior of foraging insects determines which flowers set seed and the pattern of pollen transfer (and thus male gametes) between plants, and ultimately plant population genetic structure (Goulson, 1999).

The behavior of foraging insects involves decisions when encountering a food resource according to variability of nectar traits (such as volume and concentration) and their spatial distribution (Goulson, 1999; Leiss and Klinkhamer, 2005; Cnaani et al., 2006; Dreisig, 2012). The abundance and spatial distribution of nectar available to a foraging insect at a given time is called the nectar standing crop (Galetto and Bernardello, 2005). Nectar standing crop varies widely between flowers of a plant (Keasar et al., 2008). This variability is the combined result of the nectar production rate of flowers and insect foraging activity.

Plants may be under selection to produce variable nectar resources so as to economize investment in nectar production while increasing the possibility of cross-pollination. At population level, the nectar standing crop generally has a patchy distribution: one or more highly productive plants are neighbors to others that produce less (Leiss and Klinkhamer, 2005). The same happens at the smaller scale of individuals of nectar producing species that may bear a certain number of nectarless flowers (Gilbert et al., 1991; Bailey et al., 2007). Empty flowers borne by nectar-producing individuals are an energy-saving strategy that enables the plant to save resources normally allocated to nectar production while maintaining its attraction for pollinators (Bell, 1986). Nectar standing crop variability is also revealed by the generally positive skewed distribution of nectar production by individuals, which means that there are few flowers producing a large quantity of nectar and many flowers producing a smaller amount (Gilbert et al., 1991 and references therein). Nectarless and nectar-poor flowers can be considered a case of “partial cheating” when compared to the “total cheating” of deceptive nectarless plant species, reported above.

Standing crop structure (i.e., the abundance of nectar offered and its spatial distribution) affects both the duration of visits and distance between successive visits, since pollinators move quickly to more distant patches, individuals or flowers when they encounter nectarless or nectar-poor specimens (Gilbert et al., 1991; Smithson and Gigord, 2003; Leiss and Klinkhamer, 2005; Bailey et al., 2007). Short visits and fast moves between flower patches reduces the probability of geitonogamy (self-pollination between flowers on the same plant) and the risk of inbreeding. Highly variable standing crops are therefore considered a strategy to increase the out-crossing rate and offspring fitness (Smithson and Gigord, 2003; Bailey et al., 2007; Keasar et al., 2008). Moreover, plants offering high rewards may have an emanating effect on neighbors offering small rewards (Leiss and Klinkhamer, 2005). In this way plants with low nectar production may benefit from pollinator services enhanced by the presence of high nectar producing neighbors, while saving on the cost of nectar production.

Plants may exert control over nectar standing crop by providing highly variable nectar production that in turn affects the foraging behavior of pollinators. This outcome supports the idea that plants may “manipulate” the foraging behavior of pollinators to optimize pollen flow between individuals. In this framework a manipulation hypothesis was first elaborated by Biernaskie and Cartar (2004) who reported a positive correlation between variability in nectar production rate and floral display (number of open flowers) in individual plants of nine angiosperm species. According to these authors, the increased attractiveness of a plant caused by an abundance of flowers is coupled with greater variability in nectar production rates of its flowers so as to obtain an optimal trade-off between number of visits and the length of the pollinator visitation sequence.

Nonetheless, nectar standing crop is affected by two orders of variability: variability in nectar production controlled by plants, on which further variability generated by the foraging activity of pollinators is superimposed (Goulson, 1999; Keasar et al., 2008). Pollinator-generated variation seems to have major effects on pollinator foraging, possibly overriding the effects of plant-generated variation. Pollinator-generated variability in nectar resources may thus reduce the selective benefit of plant-generated variability as a strategy to decrease geitonogamy (Keasar et al., 2008). It is also worth noting that environmental parameters (at macro- and micro-environment level) may influence nectar production, standing crop and insect activity (Pacini and Nepi, 2007), further decreasing the strength of the control exerted by plants.

It follows that plant control of pollinator behavior through modulation of variable nectar production is possible but seems quite weak. Plants may, however, use other tools to influence the feeding behavior of pollinators.

### Plants Control Foraging Behavior of Pollinators by Nectar Chemistry

A nectar trait quite recently considered when studying the effect of nectar on insect feeding behavior is its chemical composition. Floral and EFN is largely composed of sugars, usually together with other primary metabolites, such as amino acids, lipids, and proteins (Nicolson and Thornburg, 2007). Secondary metabolites (alkaloids, terpenoids, and phenols) are reported more rarely than primary metabolites, but their presence is presumed to be quite common (Nicolson and Thornburg, 2007; Roy et al., 2017; Stevenson et al., 2017). Nectar secondary metabolites include volatile compounds that impart scent to both floral and EFN, enabling insects to locate it (Raguso, 2004; Röse et al., 2006).

Both primary and secondary metabolites can have effects on insect behavior.

#### Effects of Primary Metabolites on Pollinators

Sugars and amino acids are the most abundant primary metabolites and are an important source of energy and nitrogen, respectively (Roy et al., 2017 and reference therein). They are therefore the main determinant of the food value of nectar, but they can also affect the attractiveness of nectar since they are responsible for its taste (Gardener and Gillman, 2002). Both sugars and amino acids affect insect feeding behavior through post-ingestive signaling, involved in associative learning and memory (Simcock et al., 2014, 2018), processes that are of particular importance in making choices during foraging. Associative learning is a mechanism that allows animals to identify cues associated with nutrients so that they can be located quickly when required (Simcock et al., 2014).

Sucrose is the most common and abundant nectar sugar (Baker and Baker, 1983b) and is preferred by honeybees to other naturally occurring sugars (Barker and Lehner, 1974). Its concentration is an important determinant for many foraging-related decisions (Scheiner et al., 2004). Interestingly, this disaccharide is recognized as the most phagostimulatory sugar for honeybees, and bees rewarded with sucrose are more likely to learn to associate an odor with a food source (Simcock et al., 2018).

All twenty amino acids commonly found in proteins have been identified in various plant nectars. Proline seems to be of special importance for insects. It not only contributes a taste preferred by insects (Alm et al., 1990; Bertazzini et al., 2010), but also stimulates the insect salt cell, a labellar chemosensory receptor, resulting in increased feeding behavior (Hansen et al., 1998). In an experiment using free-flying foragers, Hendriksma et al. (2014) demonstrated that honeybees preferred essential over non-essential nectar amino acids. Phenylalanine, one of the most abundant amino acids in nectar (Petanidou, 2007), has strong phagostimulatory activity, while glycine is a phagodeterrent, both at concentrations similar to that occurring naturally in nectar (Hendriksma et al., 2014). The same authors also demonstrated a trade-off between sucrose concentration and amino acid preferences: nectar with low sucrose concentration that is normally unattractive to bees can become attractive if it contains minute concentrations of the phagostimulant phenylalanine, whereas the phagodeterrence of glycine can be masked by high concentrations of sucrose (Hendriksma et al., 2014). It follows that plants can replace expensive carbohydrates in their nectar with minute concentrations of phagostimulating amino acids, or modulate pollinator visits by adding phagodeterrent amino acids.

The link between sucrose and amino acids in affecting feeding behavior was also revealed by experiments testing how nutritional state affected the taste of specific amino acids (isoleucine, proline, phenylalanine, and methionine) and associative learning of honeybees (Simcock et al., 2014). Results showed that bees pre-fed sucrose solution consumed less of solutions containing amino acids and were less likely to associate amino acid solutions with odors. Surprisingly, bees pre-fed solutions containing an amino acid were also less likely to associate odors with sucrose the next day. Bees consumed more food and were more likely to learn when rewarded with an amino acid solution if they were pre-fed isoleucine and proline (Simcock et al., 2014). The authors concluded that single amino acids at relatively high concentrations decrease feeding on sucrose solutions containing them, and they can act as appetite reinforcers during learning.

#### Effects of Secondary Metabolites on Pollinators

Plant secondary metabolites (SMs) can be defined as “compounds that do not occur universally but are restricted to specific plant taxa, or occur in certain plant taxa at much higher concentrations than in others, and have no (apparent) role in primary metabolism” (Schoonhoven et al., 2005). Plants produce a plethora of SMs with a variety of functions. They are mainly involved in defense against herbivores and other enemies such as fungi and bacteria but may also have other additional functions (Schoonhoven et al., 2005). Secondary metabolites, including tannins, phenols, alkaloids, and terpenes, have been found in FN in more than 21 angiosperm families (Adler, 2000). These compounds have been known since the 1970s and were initially considered to be toxic deterrents of nectar thieves while encouraging specialist pollinators (Baker and Baker, 1983a; Adler, 2000; Barlow et al., 2017; Stevenson et al., 2017). More recently, researchers have discovered that these compounds, and particularly alkaloids, may play an important role in managing visitor behavior.

Nicotine (a pyridine alkaloid) is a typical insect-repelling alkaloid and is found in the FN of *Nicotiana attenuata*, where it increases the number of flowers visited and reduces the volume of nectar consumed by hummingbirds and moth pollinators (Kessler and Baldwin, 2007). The unpleasant taste of nectar containing nicotine reduces nectar consumption and the length of flower visits, leading to a higher rate of outcrossing (Kessler et al., 2012). Shorter visits also reduce the risks associated with excessive visitation of individual flowers, such as increased reception of incompatible pollen or removal of compatible pollen grains from the stigma surface (Pyke, 1984). Plants with FN containing nicotine are able to minimize nectar volumes, while maximizing pollination efficiency, seed production and plant fitness. In this perspective the function of nectar is not to increase flower attractiveness but rather to optimize pollen flow between individuals by altering the feeding behavior of insects. This outcome clarifies the apparent contrast between the general deterrent effect of SMs and plants’ need to efficiently attract insects as vectors of pollen.

Other nectar SMs may have phagostimulatory activity, although this function seems restricted to species adapted to feed on plants with a high content of SMs (Stevenson et al., 2017). Note that SM effects on insects are dose dependent (Manson et al., 2013) and their concentrations in nectar may also be highly variable in a single plant; however, it is generally recognized that SM levels in nectar are lower than in other plant tissues (Cook et al., 2013).

The feeding deterrent function of nectar SMs is due to the unpalatable taste of alkaloids, especially nicotine, that is perceived by insects as soon as their proboscis contacts the nectar. The mouth parts of insects have contact chemoreceptors with neurons responding to sugars, salts, acid, water and non-nutrient compounds (Stevenson et al., 2017 and references therein). As in the case of amino acids (see above), chemoreceptor response to nectar SMs is modulated according to sucrose concentration: rejection of high concentrations of SMs can be attenuated by high carbohydrate content of nectar (Köhler et al., 2012).

Nectar SMs may have post-ingestive effects on other targets in the insect body, such as the brain, affecting their neurobiology (**Table 1**). It was recently reported that honeybees rewarded with solutions containing caffeine (a purine alkaloid) at concentrations similar to that occurring naturally in the FN of *Coffea* and *Citrus* species, remembered the learned floral scent better than honeybees rewarded with sucrose alone (Wright et al., 2013; **Table 1**). Caffeine, an adenosine-receptor antagonist, affected Kenyon cells’ activity, potentiating the response of honeybee brain mushroom body neurons that are involved in olfactory learning and memory formation (Wright et al., 2013 and references therein). At higher concentrations, caffeinated solutions exerted a deterrent effect and bees were more likely to reject caffeinated solutions. Pollinators therefore drive selection for nectar that is not repellent but still has neurobiological activity. The “increased memory” effect of nectar-like concentrations of caffeine may be one reason for unexplained flower constancy, frequently observed in foraging honeybees (Goulson, 1999). From the plant perspective, pollinator constancy is clearly beneficial since it minimizes pollen wastage and unfruitful heterospecific pollination.

**Table 1:** Secondary compounds and their hypothesized or tested post-ingestive effects on neurobiological or physiological traits of insects.

Compound	FN	EFN	Tested insect	Behavioral/physiological effects	Reference
Caffeine	×		Honeybees (*Apis mellifera*)	Increased learning and memory at nectar-level concentrations	Wright et al., 2013
Caffeine and theophylline			Ants (*Myrmica sabuleti*)	Increased linear speed, memory, and conditioning ability. Decreased consumption of food and precision of reaction.	Cammaerts et al., 2014
Nicotine	×		Bumblebees (*Bombus terrestris audax)*	Increased learning and memory at nectar-level concentrations	Baracchi et al., 2017
Cocaine			Ants (*Myrmica sabuleti*)	Increased audacity. Decreased linear speed, precision of reaction, response to pheromones and consumption of food. Inhibited conditioning ability. Induced dependence	Cammaerts et al., 2014
Atropine			Ants (*Myrmica sabuleti*)	Decreased olfactory perception and precision of reaction	Cammaerts et al., 2014
Non-protein amino acids (GABA, β-alanine)	×			Effects on muscle activity, nervous system, and phagostimulation	Nepi, 2014; Felicioli et al., 2018
Chitinase (nectar protein)		×	Ants (*Crematogaster*)	Inhibition of gut invertase	Heil et al., 2014

A similar behavioral effect was reported in bumblebees fed with solutions containing nicotine at concentrations within or above the natural range (**Table 1**). Bumblebees were only deterred by unnaturally high nicotine concentrations (50 ppm) and this deterrence disappeared or became attraction at lower nectar-like concentrations (1 and 2.5 ppm) (Baracchi et al., 2017). The same concentrations affected bumblebee flower preference through enhanced memory of floral traits. Increasing numbers of bumblebees remained faithful to flowers containing nicotine at any tested concentration, even if they become a suboptimal choice in terms of caloric value (Baracchi et al., 2017). Although the neurobiological mechanism was not studied, it is postulated that nicotine, being an agonist of nicotinic acetylcholine receptors, may act as a psychoactive drug, modulating cholinergic neuron activity in the insect brain and positively reinforcing the flower-reward association (Baracchi et al., 2017 and references therein).

In addition to alkaloids, other nectar SMs such as non-protein amino acids (NPAAs), i.e., amino acids that are not used by organisms to build proteins, are potentially involved in modulating insect behavior (**Table 1**). Those more common in nectar, i.e., γ-aminobutyric acid (GABA) and β-alanine, are important insect nervous system neuromodulators (Nepi, 2014 and references therein). They may affect insect behavior in several ways: by affecting insect nervous system physiology, regulating nectar intake through phagostimulation and promoting muscle function (Felicioli et al., 2018). Among the NPAAs found in nectar, GABA seems of particular interest since in invertebrates GABA-receptors are located peripherally in muscle tissue and neuromuscular junctions bathed in hemolymph (Bown et al., 2006) and may be sensitive to variations in GABA levels caused by insect feeding on GABA-rich nectar. However, no clear confirmation of this hypothesis has yet been found.

### Do Plants Control the Behavior of Ants by Means of EFN?

In the case of EFN, there is evidence that variations in nectar productivity between plant species and at different times of day may influence the visitation patterns of ants and in some cases also their numbers, showing the important key role of these nectaries in ant-plant interaction systems (Blüthgen et al., 2000; Lange et al., 2013, 2017). However, nectar quality and certain ant behaviors may also have important consequences for the organization and distribution of ant foraging activities (Blüthgen et al., 2000; Blüthgen and Stork, 2007; Anjos et al., 2017). Compared to FN, the effects of EFN chemistry on ants (and other predators visiting plants bearing EFNs) has certainly been neglected. It has been reported that the unbalanced C/N ratio of nectar may increase the ants’attraction for *N*-rich food, and hence the likelihood that they will attack herbivorous insects on the host plant, contributing to indirect defense of the plant (Ness et al., 2009). Thus it appears that indirect plant protection involving ants is elicited by plant-mediated dietary imbalances. Actually, the aggressiveness of tending ants increases with increasing EFN carbohydrate content (Grover et al., 2007; González-Teuber et al., 2012) but there may be another explanation. Carbohydrates are a major fuel for metabolically expensive behaviors, such as ant aggressiveness and hyperactivity. In any case, higher and lower C/N ratios have been reported in response to herbivore activity, with EFN sucrose (Ness, 2003) and amino acid (Smith et al., 1990) contents both increasing after herbivore attacks. It has also been suggested that changes in the C/N ratio of EFN could manipulate the prey preferences of foraging ants: increasing EFN carbohydrate levels resulted in reduced feeding on high lipid prey (Wilder and Eubanks, 2010).

There is little literature on secondary metabolites in EFN. Trace amounts of the alkaloid harmine were reported in EFN of *Passiflora edulis* (Cardoso-Gustavson et al., 2013). This alkaloid was retained in the extra-floral nectary at high concentrations as well as excreted into EFN at low concentrations. The plant modulated secondary metabolite concentrations to relate differently to herbivores and mutualistic consumers: high concentrations in EFNs protected the gland from herbivores while low (trace) concentrations in EFN had no apparent effect on ants (Cardoso-Gustavson et al., 2013).

Though not reported in EFN, four alkaloids (caffeine, theophylline, cocaine, and atropine) can have significant effects on many aspects of ant physiology and behavior (Cammaerts et al., 2014; **Table 1**). In particular, when ingested, the alkaloids altered locomotion, memory, olfactory perception and reactions to stimuli in the *Myrmica sabuleti* ant model (**Table 1**). Whether any of these or other neuroactive compounds could be components of EFN, and their effects on attending ants at concentrations plausible for EFN, are not known. In this context, it is worth noting that ants are subject to manipulation by other organisms (Hughes, 2012; Grasso et al., 2015). A recent case regards blue butterflies (Lepidoptera: Lycaenidae) whose caterpillars produce a sugary secretion that attracts ants which then defend the larvae from predators. Hojo et al. (2015) found that these secretions are not simply nutritious food, but also affect ant behavior, enhancing their cooperative services.

A striking case of partner manipulation involving the myrmecophyte *Acacia cornigera* and the mutualist ant *Pseudomyrmex ferrugineus* is therefore not surprising (Heil et al., 2014). These ants only feed on the sucrose-free nectar produced by their host plant; the nectar is not attractive to other generalist exploiter ants. Until a few years ago, *Pseudomyrmex ferrugineus* ants were believed to lack invertase (a sucrose hydrolysing enzyme) in their digestive tract, a physiological trait compensated by the plant through secretion of sucrose-free EFN (Heil et al., 2005). However, this “specialization” hides a clear case of partner manipulation by the host plant. In fact, invertase activity is not constitutionally absent in the ant midgut but is inhibited by chitinase (**Table 1**), a dominant EFN protein that has a primary function in defense against nectar-dwelling pathogenic fungi (González-Teuber et al., 2010). Once eclosed, young workers ingest EFN as the first food available. Since this inhibits their invertase, they are forced to continue feeding on host-derived EFN, being unable to digest any other food (Heil et al., 2014). The plant manipulates the digestive physiology of the symbiotic ants to enhance their dependence on host-derived food rewards, thus stabilizing in the partnership and avoiding possible interference by exploiters.

## Concluding Remarks and Future Challenges

**FIGURE 2:**
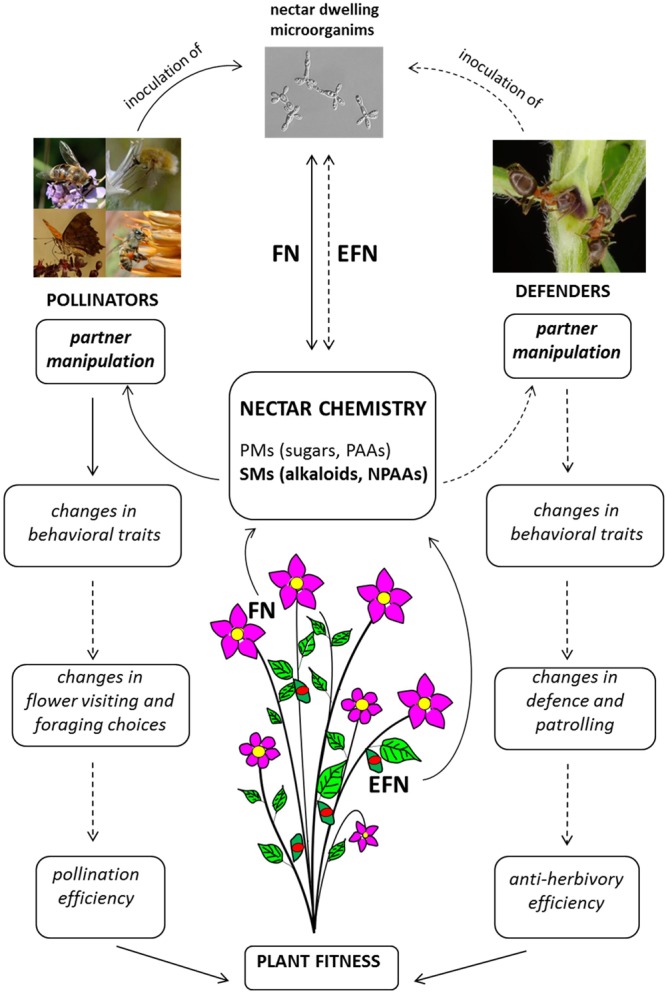
Diagram of nectar-mediated manipulation of pollinators and tending ants. Full lines indicate processes/interactions sustained by scientific evidence; hatched lines indicate processes/interactions for which scientific evidence is not yet available. FN, floral nectar; EFN, extra-floral nectar; PMs, primary metabolites; PAAs, protein amino acids; NPAAs, non-protein amino acids; SMs, secondary metabolites. Picture of nectar-dwelling microorganism (*Metschnikowia gruessii*) reproduced with permission from Carlos M. Herrera.

Recent research on nectar-mediated plant–animal interactions highlights that FN and EFN is much more than a sugary reward for animal services. As suggested by Pyke (2016), nectar can now be viewed as a pollinator manipulant rather than simply an attractant or reward (**Figure 2**). Clear effects of nectar-mediated manipulation are known for pollinating insects and are mainly based on secondary metabolites in FN (**Figure 2**). Although detailed studies are only available for caffeine and nicotine (Kessler et al., 2012; Wright et al., 2013; Baracchi et al., 2017), other known psychoactive compounds from plants could also manipulate pollinator behavior but have not yet been investigated in nectar.

The real outcomes of these manipulative strategies are not yet well-understood. Plants enhance recall of a food resource by presenting appropriate concentrations of psychoactive drugs in FN (Wright et al., 2013; Baracchi et al., 2017). This strategy may ensure pollinator fidelity and possibly improve the plant’s reproductive success, but experimental evidence is not yet available (**Figure 2**). On the animal side, although improved recall can be positive for efficient foraging activity, it also has a negative counterpart since bees tend to return to the source of caffeinated nectar when it is no longer available (Couvillon et al., 2015) and this may have negative consequences for the pollinator. It seems that “manipulated” pollinators still obtain the benefits of nectar consumption, but in the case of a net negative outcome for animal fitness, manipulation may turn a mutualism into parasitism (Heil, 2015b; Hojo et al., 2015).

The presence of nectar-dwelling microorganisms adds a further level of complexity to these manipulative interactions (**Figure 2**). Microorganisms such as yeasts and bacteria are very common in FN where they are inoculated by pollinators and can be considered a third partnership in nectar-mediated plant–pollinator interactions (Herrera et al., 2009). They are responsible for drastic changes in nectar chemical profile that potentially affect pollinator behavior and foraging choices: they alter the concentrations of specific sugars and amino acids (Canto and Herrera, 2012; de Vega and Herrera, 2013; Pozo et al., 2014; Lenaerts et al., 2016; Vannette and Fukami, 2018) and produce volatile substances that are perceived by pollinators (Raguso, 2009). Interestingly, microorganisms are also able to alter the profile of nectar SMs. For example, they can significantly lower the concentration of nicotine and thus interactions with pollinators, since the effects of secondary compounds are concentration-dependent (Vannette and Fukami, 2016).

Ants are known to transport microorganisms (de Vega and Herrera, 2013) although the presence of the latter in EFN has never been reported.

Another aspect that needs to be considered in reporting complex outcomes of manipulative exploitation in mutualistic relationships is that nectar is a complex mixture of solutes, while experiments on the effects of nectar-specific compounds are often conducted on single molecules, ignoring any synergic or antagonistic effects.

Secondary metabolites in EFN and their possible interactions with tending ants (and other insects) have not been the subject of much research (**Figure 2**). Complexity similar to that of FN-mediated interactions is also likely for EFN but has not yet been investigated (Grasso et al., 2015). Since the targets of indirect defense by mutualism with ants are plant enemies such as herbivores, aggression is an obvious ant behavioral trait that could be manipulated by plants, although other less conspicuous behaviors could also be affected and have significant positive effects (Grasso et al., 2015).

Plants modulating the concentration of SMs in their tissues and secretions evolved strategies to deter herbivores (high concentrations), while attracting and manipulating mutualists (low concentrations) to maximize the benefits they obtained. When such strategies evolved is hard to say. The oldest plant–insect relationship is predation of plants by herbivores and plants underwent natural selection on the basis of chemical defenses (secondary metabolites) evolved against herbivores. When mutualistic insects evolved (defenders and pollinators) they presumably drove plant selection toward optimal (low) concentrations of SMs (and other substances) in secretions they fed on, while plants probably started to manipulate insect behavior pharmacologically, improving their own fitness. Most “modern” mutualist insects (Diptera, Lepidoptera, and Hymenoptera including ants) radiated in the interval 125–90 Mya (i.e., early-middle Cretaceous), simultaneously with angiosperms (Labandeira, 2011). Nectars with SM profiles presumably evolved and diversified in angiosperms and allowed them more efficient interactions with insects, overriding interactions already established by gymnosperms (Nepi et al., 2017).

Concluding, since conflicts also arise in cooperative partnerships, nectar-mediated partner manipulations may be more frequent than previously thought in plant–insect interactions conventionally regarded as mutualistic. This may provide new evidence supporting the idea that elements of coercion/manipulation are not necessarily linked to parasitic habits but may be functional for stabilizing certain insect–plant mutualisms (Heil, 2015b), opening new horizons in the study of coevolutionary pathways involving these dominant organisms.

## Author Contributions

MN, DG, and SM conceived the idea of the article and wrote the final version of the paper. MN designed the outline and wrote the draft of the paper. DG and SM commented on the draft.

## Conflict of Interest Statement

The authors declare that the research was conducted in the absence of any commercial or financial relationships that could be construed as a potential conflict of interest.
